# Nonclinical pharmacokinetics and activity of etirinotecan pegol (NKTR-102), a long-acting topoisomerase 1 inhibitor, in multiple cancer models

**DOI:** 10.1007/s00280-014-2577-7

**Published:** 2014-09-17

**Authors:** Ute Hoch, Carl-Michael Staschen, Randall K. Johnson, Michael A. Eldon

**Affiliations:** 1 Nektar Therapeutics, 455 Mission Bay Boulevard South, San Francisco, CA 94158 USA; 2 Food and Drug Administration, Rockville, MD USA; 3 Independent Consultant, Santa Fe, NM USA

**Keywords:** Topoisomerase 1 inhibition, Anticancer activity, Etirinotecan pegol, SN38, Pharmacokinetics

## Abstract

**Purpose:**

The aim of the study was to demonstrate the activity of etirinotecan pegol, a polymer conjugate of irinotecan, in multiple human tumor models and to establish both the pharmacokinetic/pharmacodynamics (PK/PD) relationship and clinical relevance of the findings.

**Experimental design:**

Anti-tumor activity was evaluated in mouse models of human lung, colorectal, breast, ovarian, and gastric cancers. Etirinotecan pegol was administered intravenously (once or every 3–7 days) to animals with established tumors. Activity was assessed by tumor growth delay (TGD) and regression. Mice bearing established colorectal and lung tumors were treated with etirinotecan pegol or irinotecan, and serial blood and tumor samples were collected at planned times between 0 and 60 days post-treatment for quantitation of etirinotecan pegol and SN38. For PK analysis, analyte concentration–time data were fit with compartmental models; PK/PD analysis was based on an inhibitory *E*_max_ response model.

**Results:**

Etirinotecan pegol was active in all tumor models. TGD was sustained for 2–10 weeks after last dose, while conventional irinotecan resulted in little suppression of tumor growth. Etirinotecan pegol was eliminated very slowly from the tumor (*t*_1/2_ = 17 days), achieving higher and more sustained tumor exposure when compared with conventional irinotecan. The increased tumor exposure following etirinotecan pegol correlated with strong and prolonged suppression of tumor growth. Sustained plasma exposure to active SN38 was consistently observed across nonclinical species (including mouse, rat, and dog) and translated to cancer patients.

**Conclusions:**

Etirinotecan pegol is the first long-acting topoisomerase 1 inhibitor that provides sustained exposure, which results in prolonged anti-tumor activity in a wide variety of cancer models.

**Electronic supplementary material:**

The online version of this article (doi:10.1007/s00280-014-2577-7) contains supplementary material, which is available to authorized users.

## Introduction

The application of nanotechnology and polymer chemistry to improve chemotherapy is currently an active field of cancer research [1]. Nanoparticle chemotherapeutics aim to increase activity and improve the safety of conventional chemotherapeutics by trafficking a greater fraction of administered drug directly to cancer cells in a controlled fashion. Polyethylene glycol (PEG)ylated liposomal doxorubicin (Doxil^®^), liposomal daunorubicin (DaunoXome^®^), liposomal cytarabine (DepoCyt^®^), and paclitaxel-bound particles (Abraxane^®^) are members of this class of agents approved in the USA [2–5] for the treatment of solid tumors and hematological malignancies [6, 7]. Conjugation with PEG has also emerged as an effective technology for extending exposure to active agents and improving the pharmacokinetics (PK) and reducing the immunotoxicity of proteins. Its application to small molecules is actively being pursued [8, 9].

**Fig. 1:**
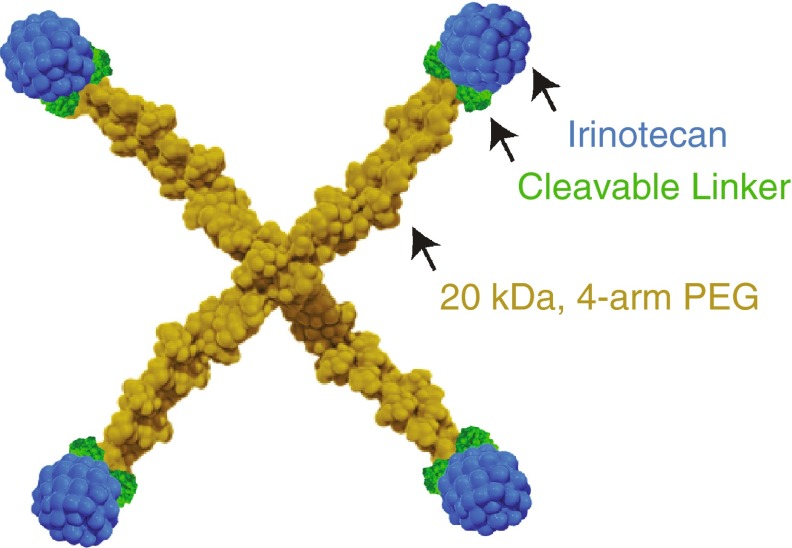
Schematic structure of etirinotecan pegol

Etirinotecan pegol (NKTR-102) is a long-acting polymer conjugate of irinotecan, a topoisomerase 1 (Top1) inhibitor, designed to provide continuous exposure of SN38 in tumors while avoiding high irinotecan and SN38 plasma concentrations that lead to high AUCs associated with unwanted side effects [10]. Etirinotecan pegol uses proprietary polymer conjugation with large-chain polyethylene glycols (PEGs) to enhance PK and pharmacodynamic (PD) characteristics of its active moiety. Schematically, etirinotecan pegol consists of a 4-arm PEG polymer with a nominal molecular weight of 20 kDa, a cleavable ester-based linker, and one irinotecan molecule at the end of each arm (Fig. 1). Upon administration, the cleavable linker in etirinotecan pegol slowly hydrolyzes, resulting in sustained exposure to irinotecan that is subsequently metabolized into the active metabolite SN38.

The irinotecan pharmacophore of etirinotecan pegol is the active pharmaceutical ingredient of Camptosar^®^ (camptothecin-11; CPT-11), a Top1 inhibitor that is widely used as a chemotherapeutic agent [11]. CPT-11 is indicated for the treatment of colorectal cancer in combination with 5-fluorouracil (5-FU) and folinic acid (first line) and as a single agent in patients who progressed following initial 5-FU-based therapy (second line) [12–14]. Top1 inhibition with irinotecan has also demonstrated clinical benefits in the treatment of small cell lung cancer [15], non-small cell lung cancer [16], esophageal cancer [17], gastric cancer [18], central nervous system cancers [19], cervical cancer [20], breast cancer [21], and non-Hodgkin’s lymphoma [22]. The primary mode of action is through inhibition of the Top1 enzyme, resulting in the formation of covalent and nonreversible Top1-DNA complexes that are converted into lethal DNA lesions when the DNA replication fork collides with this stabilized complex [23]. Irinotecan is a prodrug that is activated via enzymatic cleavage of the C-10 side chain by carboxylesterases (CES) to generate the biologically active metabolite, 10-hydroxy-7-ethyl camptothecin or SN38, which has 100- to 1,000-fold more potent cytotoxicity in vitro compared with irinotecan [24].

Although irinotecan has clinical utility, its anti-tumor activity may be limited by the short half-life due to inactivation at physiological pH by the opening of its lactone E-ring and rapid clearance of the parent drug and its active metabolite SN38. In humans, the terminal half-life (*t*_1/2_) of irinotecan is 9–14 h [25, 26], while the *t*_1/2_ of SN38 is 24–47 h [26, 27]. The recommended irinotecan dose and schedule of 350 mg/m^2^ every 21 days results in high peak plasma concentrations near the end of infusion. When administered as protracted infusions instead of short infusions, the toxicity profile of irinotecan changes, with lower incidences of cholinergic reactions and severe myelosuppression [28–30], suggesting that high peak plasma concentrations contribute to those known toxicities. Following infusion, concentrations of irinotecan and SN38 fall below 0.1 ng/mL 5–7 days post-dose (SN38 concentrations less than 0.1 ng/mL have been reported for up to 500 h in one study [26]), resulting in an absence of drug exposure until the next dose is administered. This short and intermittent exposure could limit the effectiveness of the anti-tumor activity of irinotecan [21, 29].

Etirinotecan pegol was selected based on reduced peak plasma concentrations, prolonged tumor exposure, and improved biodistribution of irinotecan and SN38 to tumor sites. Using xenograft mouse models of human cancers, a library of irinotecan analogs with varying PEG sizes, architectures, and linkers was studied, leading to the identification of etirinotecan pegol. Here, we report the results from a series of studies comparing the in vivo performance of etirinotecan pegol and conventional irinotecan, including evaluation of PK and PK/PD, which was tested in multiple animal species (mouse, rat, and dog), and activity in mouse xenograft models of human colorectal, non-small cell lung, breast, gastric, and ovarian cancers. The promising results from these studies lead to the selection and Phase 2 clinical evaluation of etirinotecan pegol for advanced breast, ovarian, colorectal, small cell lung cancer, non-small cell lung cancers, and glioblastoma as well as subsequent Phase 3 evaluation for advanced breast cancer.

## Materials and methods

### Chemicals and reagents

Irinotecan (Camptosar^®^ 20 mg/mL solution, Pharmacia & Upjohn Company) was diluted to appropriate concentrations with normal saline in subdued light immediately prior to injection.

Etirinotecan pegol (Nektar Therapeutics) was dissolved in saline or 5 % dextrose in water to make a stock solution and then further diluted with saline or 5 % dextrose in water to achieve appropriate injection volumes (0.2 mL/10 g body weight in mice; 4 mL/kg for rat; 5 mL/kg for dog). All doses and plasma tumor concentrations of etirinotecan pegol are expressed based on irinotecan content, which enables a direct comparison with irinotecan.

### Animal studies

Female athymic nude mice (Ncr:Nu and Nu:Nu), male Sprague–Dawley rats, and male Beagle dogs were purchased from Charles River Laboratories or Harlan Laboratories. The mice were used for HT29, NCI-H460, MCF-7, and A2780 xenograft studies. Female severe combined immunodeficient mice (CB.17/Icr-Prkdc^scid^) were purchased from Charles River Laboratories and used for the NCI-N87 study. Mice were housed in microisolator cages with a 12-h light/dark cycle and received sterilized food and water ad libitum. Etirinotecan pegol and irinotecan were administered as an intravenous bolus in mice, 30-min intravenous infusion in rats, and 1-h intravenous infusion in dogs.

### Pharmacokinetic and pharmacodynamic study in tumor-bearing mice

Tumor fragments (30–40 mg) of either human HT29 colon or NCI-H460 lung tumor were implanted subcutaneously near the right axillary area. Tumors were allowed to reach a median volume of 100–172 mm^3^ for HT29 tumors (13 days) and 100–245 mm^3^ for NCI-H460 (9 days) prior to the start of dosing. Animals were randomized into groups of 4 mice per sampling time and given 40-mg/kg irinotecan-equivalent intravenous bolus doses of etirinotecan pegol or irinotecan on Days 0, 4, and 8. The length and width of each tumor was recorded 2–3 times a week, and the corresponding tumor volume was estimated using the formula *L* × *W* ^2^/2 = mm^3^, where *L* and *W* refer to the larger and smaller perpendicular tumor dimensions, respectively. Animals found moribund or with tumors ≥4,000 mm^3^, ulcerated, or sloughed off were euthanized prior to scheduled termination. Blood and tumor tissue samples were obtained using the following schedules: HT29 tumor-bearing mice: before dosing and 0.5, 1, 4, 8, and 12 h and 1, 2, 3, 4, 5, 10, 12, 15, 20, 30, 40, 50, and 60 days after the start of dosing; NCI-H460 tumor-bearing mice: before dosing and 0.5, 1, 4, and 12 h and 1, 3, 5, 10, 12, 15, 20, 25, and 30 days after the start of dosing. Blood samples were collected via retro-orbital sinus sampling into blood collection tubes containing 3 mg of sodium fluoride and 6 mg of Na_2_EDTA and kept on ice until centrifuged at 2,100×*g* for 20 min at 4 °C. Plasma was harvested, frozen on dry ice, and stored at −80 °C until assayed. Immediately following blood collection, animals were euthanized, and the tumors were excised, frozen in liquid nitrogen, and stored at −80 °C until assayed.

### Pharmacokinetic studies in rats and dogs

Blood samples were collected via a peripheral vein into blood collection tubes containing 1/20th the plasma volume of a dimethyl sulfoxide solution containing 50 mM phenylmethyl sulfonyl fluoride and 1 % acetic acid (v/v). Plasma was harvested by centrifugation at 2,100×*g* for 20 min at 4 °C. Plasma was further stabilized by addition of 1 % acetic acid and stored at −80 °C until assayed.

### Assay of drug and metabolites in plasma and tumor

Plasma and tumor samples were assayed for etirinotecan pegol, irinotecan, and SN38 using liquid chromatography–tandem mass spectrometry (LC–MS/MS) methods. Tumor tissue samples were homogenized in a 9× volume of homogenization buffer (containing sodium fluoride, phenylmethyl sulfonyl fluoride, and sodium dodecyl sulfate) prior to extraction of analytes. SN38 from tumor homogenate was extracted using protein precipitation with acetonitrile and quantified by LC–MS/MS using SN38 calibration standards. LC–MS/MS used a Synergy Hydro, 3 µm, 50 × 2.0-mm column, operated at 50 °C, at a flow rate of 0.4 mL/min with a gradient consisting of 0.1 % formic acid in water and 0.1 % formic acid in acetonitrile, coupled to an API 4000 (Applied Biosystems). Irinotecan and SN38 from rat and dog plasma samples were extracted using protein precipitation with acetonitrile followed by liquid–liquid extraction with methyl tertiary butyl ether. LC–MS/MS used an Onyx Monolithic C18, 3 µm, 100 × 3-mm column, operated at 30 °C, at a flow rate of 1–2.5 mL/min with a gradient consisting of 0.1 % formic acid in water and 0.2 % formic acid in 75:25 acetonitrile/methanol coupled to an API 4000 (Applied Biosystems). Etirinotecan pegol was extracted from separate aliquots using solid phase extraction (mice) or protein precipitation with acetonitrile (rat and dog). For mice, etirinotecan pegol was then hydrolyzed (pH 7, 90 °C for 2 h) to release irinotecan, which was quantified by LC–MS/MS, using calibration standards containing etirinotecan pegol and irinotecan. LC–MS/MS used a Synergy Hydro 3 µm, 50 × 2.0-mm column, operated at 50 °C, at a flow rate of 0.4 mL/min with a gradient consisting of 0.1 % formic acid in water and 0.1 % formic acid in acetonitrile, coupled to an API 4000 (Applied Biosystems). For rat and dog plasma samples, supernatant from protein precipitation containing etirinotecan pegol was directly quantified by LC–MS/MS, using calibration standards consisting of etirinotecan pegol. LC–MS/MS used an Intrada WP-RP, 50 × 2.0-mm column, operated at 60 °C, at a flow rate of 0.5–1.0 mL/min with a gradient consisting of 0.8 % formic acid in water and 0.8 % formic acid in acetonitrile, coupled to an API 4000 (Applied Biosystems). The lower limits of quantitation for etirinotecan pegol were 1.2 μg/mL in mouse plasma, 0.01 μg/mL in rat and dog plasma, and 12 μg/g in tumor homogenate. The lower limits of quantification for irinotecan and SN38 were 0.001–0.003 and 0.0002–0.003 μg/mL in plasma, and 0.030 and 0.030 μg/g in tumor homogenate, respectively.

### Plasma and tumor pharmacokinetics of etirinotecan pegol, irinotecan, and SN38

Mean mouse plasma and tumor concentration–time data for each analyte were fit with one- or two-compartment PK models [31], as dictated by each dataset, using Berkeley Madonna, version 8.3.18 (University of California in Berkeley), to predict a concentration–time profile for the duration of each study. The predicted mouse and measured rat and dog concentration–time profiles were analyzed with noncompartmental methods within WinNonlin (Professional version 5.2; Pharsight Corporation, Mountain View, CA) to estimate customary PK parameters.

### Tumor pharmacokinetic and pharmacodynamic (PK/PD) analysis

Inhibitory *E*_max_ response PK/PD models based on the Gompertz equation [32] were developed for both tumor types to represent the rate of tumor growth as a function of tumor SN38 concentration. A schematic diagram of the PK/PD model is shown in Online Resource 1. Nonlinear regression analyses were used to simultaneously fit the mean relative tumor volume versus time data for control, irinotecan, and etirinotecan pegol treatment groups. For each tumor type, tumor SN38 PK parameters were fixed at values obtained from the PK modeling described above, and maximum effect (*E*_max_ *)* was fixed at a value of 1. The PD parameter values *k*_*gr*_ (tumor intrinsic growth rate; day^−1^), *Limit* (limit of tumor growth), and *EC*_*50*_ (half-maximal effective inhibitory concentrations; μg/g) were optimized using Runge–Kutta 4 integration and the multiple-fit curve fitting module of Berkeley Madonna.

### Activity in mouse xenograft models

Female mice, aged 6–10 weeks and weighing 14–28 g, were used for the xenograft studies. For the HT29 and NCI-H460 models, tumor fragments from serial passages were implanted subcutaneously near the right axillary area. For the MCF-7 model, approximately 1 × 10^6^ MCF-7 cells suspended in 50 % Matrigel™ were injected subcutaneously into the right flank. Two days prior to tumor implantation, a 1-mg 17β-estradiol pellet (Innovative Research of America) was implanted subcutaneously. For the A2780 model, 1 × 10^7^ A2780 tumor cells suspended in phosphate buffered saline were injected subcutaneously into the right flank. For the NCI-N87 model, 1 × 10^7^ NCI-N87 cells suspended in 50 % Matrigel™ were injected subcutaneously into the right flank.

Tumors were allowed to reach a volume of 135–184 mm^3^ for HT29 (Day 13 post-implant), 120–171 mm^3^ for NCI-H460 (Day 8 post-implant), 50–129 mm^3^ for MCF-7 (Day 14 post-implant), 75–196 mm^3^ for A2780 (Day 14 post-implant), and 108–196 mm^3^ for NCI-N87 (Day 14 post-implant) prior to randomization into groups of 10 animals at the start of treatment. The highest dose of irinotecan administered was based on prior maximum tolerated dose (MTD) studies in each xenograft model. Etirinotecan pegol was administered at dose levels equivalent to irinotecan MTD or 100 mg/kg. Animals were monitored for toxicity by assessing average percentage weight change with the occurrence of toxicity defined as 10 % or more of animals in a given treatment group showing 20 % or more body weight loss and/or mortality. Tumor volume was estimated as described above. The median time to reach endpoints was determined: 1,500 mm^3^ for NCI-H460, 1,000 mm^3^ for HT29, 700 mm^3^ for MCF-7, 2,000 mm^3^ for A2780, and 800 mm^3^ for NCI-N87 tumors. Animals with MCF-7 tumors that lost their estradiol pellet were excluded from determination of the median time to endpoint (TTE). Treatment outcome was evaluated by tumor growth delay (TGD) and regression rates. TGD was defined as the increase in the median TTE in each treatment group compared with the control group (TC) and expressed in days. Partial regression was defined as a reduction to ≤50 % of starting tumor volume for three consecutive measurements. A complete regression was defined as a reduction in tumor volume to below measureable size (<3 × 3 mm) for three consecutive measurements.

Animals exhibiting poor condition due to tumor progression were euthanized. Studies were terminated on Days 59 (HT29), 61 (NCI-H460), 72 (MCF-7), 60 (A2780, q7dx3), 76 (A2780 qdx1), and 84 (NCI-N87).

### Statistical and graphical analyses

The logrank test was used to analyze the significance of the differences between the TTE values of treated and control or etirinotecan pegol and irinotecan treated groups. Kaplan–Meier plots were constructed to show the percentage of animals remaining in the study to perform the logrank test. Median tumor growth curves show group median tumor volumes as a function of time. When an animal exited the study due to tumor size, the final tumor volume recorded for the animal was included with the data used to calculate the group median tumor volume at subsequent time points. Prism (GraphPad) for Windows version 5.04 was used for all graphic presentation and statistical analyses.

## Results

### Etirinotecan pegol leads to sustained plasma and tumor exposure in HT29 colon and NCI-H460 lung carcinoma models

**Fig. 2:**
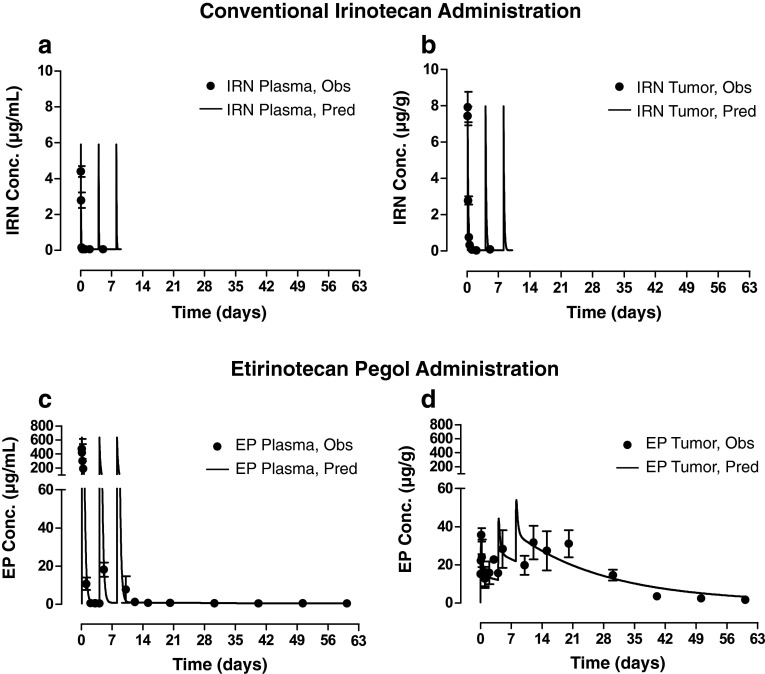
Observed and model-predicted plasma and tumor concentration–time profiles (±SEM) after intravenous administration of three doses (Days 0, 4, 8) of conventional irinotecan and etirinotecan pegol to HT29 tumor-bearing mice. **a**, **b** After administration of conventional irinotecan, plasma (**a**) and tumor (**b**) irinotecan concentrations rapidly declined to below measurable concentrations within 12 h of dosing. **c** After administration of etirinotecan pegol, plasma etirinotecan pegol concentrations also declined rapidly; however, the decline was less rapid than that observed for irinotecan, and concentrations remained measurable throughout each dosing interval and for the duration of the study. **d** In contrast to plasma, etirinotecan pegol tumor concentrations continued to accumulate with each dose, reached a maximum after the last administration, and was followed by a slow decline. Starting 24 h after each dose, etirinotecan pegol concentrations in the tumor exceeded those in plasma, consistent with tumor targeting through the enhanced permeation and retention effect. IRN, irinotecan; EP, etirinotecan pegol; *N* = 4 animals/timepoint. Etirinotecan pegol and irinotecan were administered as an intravenous bolus at 40-mg/kg irinotecan equivalents. Symbols, mean observed concentration values; solid lines, model-predicted concentration values

After administration of irinotecan, a one-compartment PK model adequately described the rapid elimination of irinotecan from plasma (*t*_½_ = 1 h), as well as the rapid appearance in and elimination of irinotecan from tumor in both models (Fig. 2 for HT29; Online Resource 2 for NCI-H460). Although tumor irinotecan AUC was ~fourfold higher compared with plasma (4.2 vs. 1.2 µg h/mL), irinotecan concentrations in both plasma and tumor were undetectable within 12 h after each irinotecan dose. In contrast, etirinotecan pegol concentrations were sustained after administration in both plasma and tumor throughout the 60-day (HT29) or 30-day (NCI-H460) study period. A three- and two-compartment PK model well described the concentrations of etirinotecan pegol in plasma and tumor, respectively. Administration of etirinotecan pegol resulted in a 400-fold and 200-fold greater plasma etirinotecan pegol AUC compared with the irinotecan AUC when administered as conventional irinotecan in the HT-29 and NCI-H460 tumor models, respectively (520 or 250 vs. 1.2 µg day/mL). Unlike administration of conventional irinotecan, the initial disposition phase for etirinotecan pegol (all etirinotecan pegol concentrations and PK parameters derived thereof are expressed based on irinotecan content to allow for a direct comparison with unconjugated irinotecan) was much slower in the tumor compared with plasma, as evidenced by its continued accumulation with each administration and higher concentrations in tumor compared with plasma. This pattern of higher concentrations in tumor compared with plasma was maintained after the last administration until the end of the study, indicating the potential for sustained release of SN38 within the tumor. Tumor etirinotecan pegol *C*_max_ concentrations were 6 and 10 times higher compared with tumor irinotecan *C*_max_ concentrations when administered as conventional irinotecan in HT29 and H460 tumors. These higher and sustained etirinotecan pegol concentrations resulted in AUC(0-60d) values that were 200-fold greater (HT29) and AUC(0-30d) values that were 140-fold greater (NCI-H460) compared with conventional irinotecan administration (860 and 600 µg day/g vs. 4.2 µg day/g).

### Etirinotecan pegol accumulation in tumor leads to high tumor SN38 exposure that correlates with marked inhibition of tumor growth

**Fig. 3:**
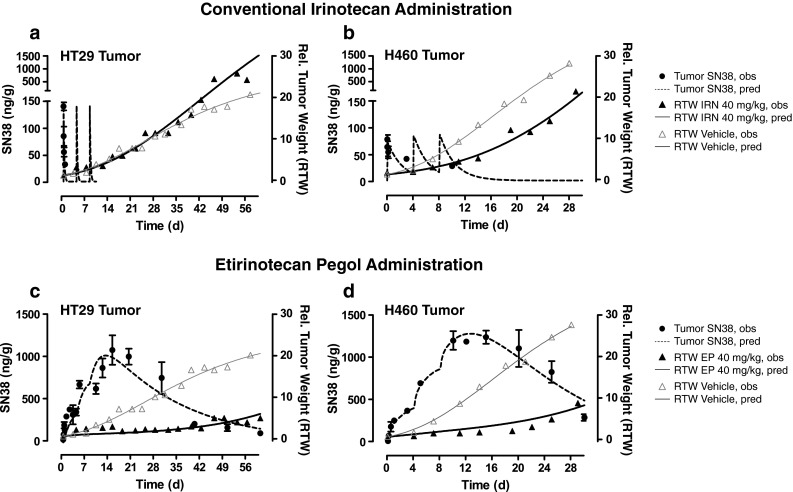
PK/PD relationship of tumor SN38 concentration (±SEM) and tumor volume after intravenous administration of conventional irinotecan, etirinotecan pegol, and vehicle to tumor-bearing mice. **a**, **b** After administration of conventional irinotecan, tumor SN38 concentrations (*circles*) rapidly declined to low or unmeasurable values, with lack of tumor growth suppression (*solid triangles*) compared with vehicle (*open triangles*) in HT29 (a) and H460 (b) tumor models. **c**, **d** After administration of etirinotecan pegol, tumor SN38 concentrations (*circles*) continued to accumulate for several days after the last dose, leading to sustained tumor growth suppression (*solid triangles*) compared with vehicle (*open triangles*) in the HT29 (**c**) and H460 (**d**) tumor models. *N* = 4 animals/timepoint. Conventional irinotecan, etirinotecan pegol, and vehicle were administered as an intravenous bolus at 40-mg/kg irinotecan equivalents on Days 0, 4, and 8. *Symbols*, mean observed values; *dotted lines*, model-predicted concentration values; *solid lines*, model-predicted tumor volumes

To verify that the tumor-localizing capability of etirinotecan pegol also translates to tumor localization of SN38, we measured intratumoral SN38 concentrations in HT29 and H460 tumors. Tumor SN38 PK mirrored those of their respective parent drug: SN38 derived from conventional irinotecan became undetectable within 12 h of each irinotecan administration, while SN38 derived from etirinotecan pegol continued to accumulate with each administration and remained above 100 ng/g until the last day of study (Fig. 3). Etirinotecan pegol delivered 300 times more SN38 to HT29 tumors (31 vs. 0.100 µg day/g) and 260 times more SN38 to NCI-H460 tumors (26 vs 0.110 µg day/g) compared with conventional irinotecan at an equivalent dose and schedule.

We next investigated the relationship between tumor SN38 exposure and tumor growth suppression. One- and two-compartment PK models coupled with an inhibitory *E*_max_ response model well described the time courses of tumor SN38 concentrations and resulting effects on tumor growth for conventional irinotecan and etirinotecan pegol, respectively (Fig. 3). Administration of etirinotecan pegol caused sustained suppression of tumors, while administration of conventional irinotecan failed to delay (HT29) or only transiently delayed (H460) tumor growth. Tumor volumes at Day 60 had increased by only sixfold after etirinotecan pegol (compared with a 30-fold increase after conventional irinotecan) in the HT29 model, even though the last dose was administered 52 days earlier. Similarly, in the NCI-H460 model, tumor volumes at Day 30 had increased by only eightfold after etirinotecan pegol, even though the last dose was administered 16 days earlier. Following conventional irinotecan treatment, there was a ≥21-fold increase in tumor volume over the same period. Tumor SN38 concentrations correlated well with the marked inhibition of tumor growth. Estimates for PK/PD parameters *k*_*gr*_, *Limit*, and *EC*_*50*_ were as follows: 0.033 (day^−1^), 52, and 0.20 µg/g for HT29; and 0.033 (day^−1^), 150, and 1.2 µg/g for NCI-H460, respectively. Tumor SN38 concentrations of this magnitude were not achieved or maintained after conventional irinotecan administration, whereas etirinotecan pegol administration resulted in high and sustained tumor SN38 concentrations that exceeded the required inhibitory concentrations for weeks after administration of the last dose.

### Etirinotecan pegol leads to increased and sustained anti-tumor activity compared with conventional irinotecan in a wide variety of tumor models

**Fig. 4:**
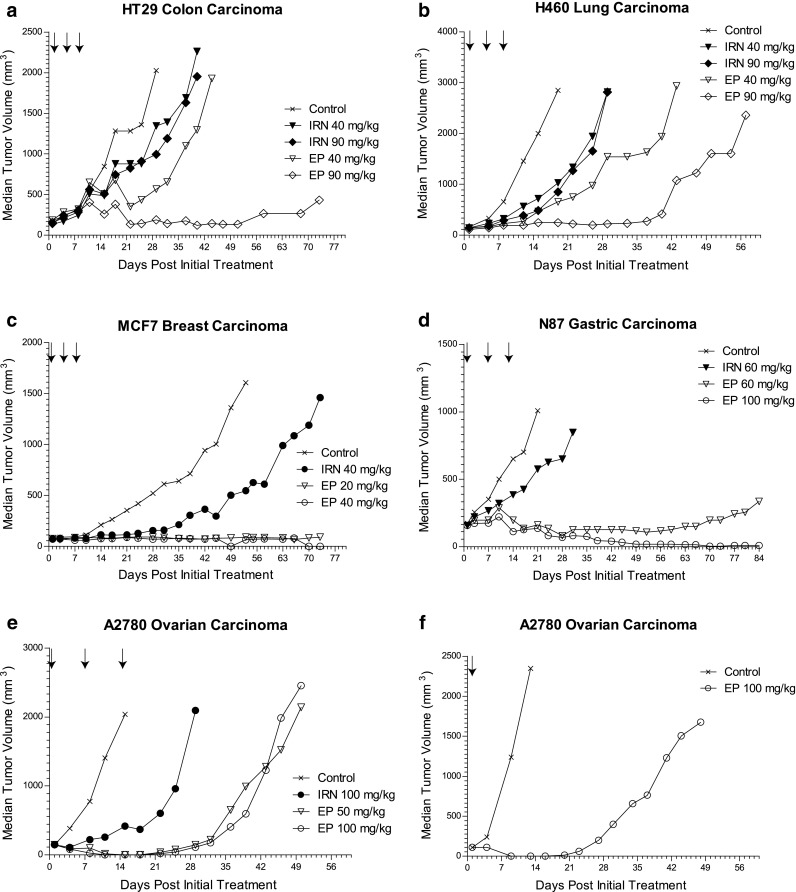
Median tumor volume versus time following treatment with etirinotecan pegol or conventional irinotecan in tumor-bearing mice. Etirinotecan pegol (EP) and conventional irinotecan (IRN) were administered as an intravenous bolus at the indicated doses (number in legend) and schedules (*arrows*). All doses are expressed as the amount of irinotecan administered. *Symbols*, median values per treatment group. *N* = 8–20 animals per treatment group

To assess whether the superior PK observed in the HT29 and NCI-H460 tumor models translated into broad antitumor activity, we compared activity of etirinotecan pegol and conventional irinotecan in a broad range of mouse tumor models. Median tumor volumes over time for all models are depicted in Fig. 4. Activity parameters are summarized in Table 1. The highest conventional irinotecan dose given was the maximum tolerated dose for the respective model; etirinotecan pegol was dosed at matching doses up to the irinotecan maximum tolerated dose or capped at 100 mg/kg. All doses of both drugs were well tolerated, with no treatment-related deaths and with maximum body weight losses of ≤11 % for etirinotecan pegol and ≤16 % for conventional irinotecan. In the HT29 colon, H460 lung, and MCF-7 breast tumor models, 3 doses of either compound were administered every 4 days starting at 13, 8, and 14 days post-tumor inoculation, respectively. Control tumors grew progressively and reached the tumor volume endpoints in 16 (HT29), 12 (H460), and 73 (MCF-7) days. The highest dose of etirinotecan pegol sustained tumor growth suppression in all three models. HT29 tumors increased only 2.7-fold, and 9 of 10 animals did not reach the tumor endpoint by the end of the study on Day 77. H460 tumors treated with high doses of etirinotecan pegol eventually started to grow on Day 37 but did not reach the endpoint by Day 56. In the breast tumor model, etirinotecan pegol suppressed tumor growth throughout the duration of the study at both doses tested, with none of the animals reaching their endpoint. In addition to sustained tumor growth delay, we observed regression responses in 20 % (HT29), 10 % (H460), and 67 % (MCF-7) of animals. In contrast, conventional irinotecan administration resulted in little tumor growth suppression and no tumor regression in the HT29 and H460 models. Although conventional irinotecan showed a significant tumor growth delay in the breast tumor model, the regression rate was ≤30 %, and 80 % of animals reached the tumor volume endpoint.

**Table 1:** Regressions and tumor growth delay for HT29, NCI-H460, MCF-7, NCI-N87, and A2780 tumor-bearing mice

Treatment	Dose^a^ (mg/kg)	Tumor regression^b^		Survivors not reaching tumor endpoint^c,d^	Median TTE^e^ (days)	TGD^f^ (days)	Statistical significance (*p*-value)	
		Partial	Complete				vs. Control	vs. High dose irinotecan
*HT29 colon tumor model*								
Control	0	0/20	0/20	0/20	16			
Etirinotecan pegol Q4dx3	40	0/10	0/10	1/10	36	20	<0.001	<0.01
	90	0/10	2/10	9/10	>60	>44	<0.001	<0.001
Irinotecan Q4dx3	40	0/10	0/10	0/10	26	10	<0.05	
	90	0/10	0/10	0/10	27	11	ns	
*NCI*-*H460 lung tumor model*								
Control	0	0/20	0/20	0/20	12			
Etirinotecan pegol Q4dx3	40	0/8	0/8	0/8	28	16	<0.001	<0.01
	90	0/9	1/9	1/9	48	36	<0.001	<0.001
Irinotecan Q4dx3	40	0/10	0/10	0/10	23	11	<0.001	
	90	0/10	0/10	0/10	24	12	<0.001	
*MCF*-*7 breast tumor model*								
Control	0	0/8	0/8	2/8	37			
Etirinotecan pegol Q4dx3	20	0/8	3/8	5/8	>73	>36	<0.01	<0.01
	40	0/8	6/9	9/9	>73	>36	<0.01	<0.01
Irinotecan Q4dx3	40	0/10	3/10	2/10	59	22	ns	
*NCI*-*N87 gastric tumor model*								
Control	0	0/9	0/9	0/9	18			
Etirinotecan pegol Q7dx3	60	2/10	1/10	10/10	>84	>66	<0.001	<0.001
	100	4/10	6/10	10/10	>84	>66	<0.001	<0.001
Irinotecan Q7dx3	60	0/10	0/10	0/10	30	12	<0.001	
*A2780 ovarian tumor model*								
Control	0	0/10	0/10	0/10	14			
Etirinotecan pegol Q7dx3	50	5/10	5/10	3/10	48	34	<0.001	<0.001
	100	2/10	8/10	3/10	46	32	<0.001	<0.001
Etirinotecan pegol Qdx1	100	0/10	10/10	1/10	44	32	<0.001	nd
Irinotecan Q7dx3	100	0/10	0/10	0/10	29	15	<0.001	

*ns* not significant, *nd* not determined

^a^All doses are expressed as amount of irinotecan administered

^b^Tumor regression must be evident for three consecutive measurements to be so designated. Partial: ≤50 % of Day 1 volume; Complete: not palpable

^c^Endpoint for HT29 was tumor volume of 1,000 mm^3^ or Day 73; for NCI-H460 was 1,500 mm^3^ or Day 57; for MCF-7 was 700 mm^3^ or Day 73; for NCI-N87 was 800 mm^3^ or Day 84; and for A2780 was 2,000 mm^3^ or Day 60

^d^Number of animals surviving through the end of study without reaching tumor endpoint

^e^Median number of days to reach tumor endpoint

^f^Tumor growth delay in treatment group

The NCI-N87 gastric and A2780 ovarian tumor models employed a weekly administration schedule, starting 14 days post-tumor inoculation. Both models grew aggressively and achieved their endpoints in a median of 18 (N87) and 14 (A2780) days. Weekly etirinotecan pegol achieved sustained tumor growth suppression in both models. In the gastric tumor model at the 100-mg/kg dose level, no tumor growth was observed during the 12-week period between start of dosing and end of the study. In fact, 50 % of animals had no tumor remaining, while the average tumor volume for the other 50 % of animals decreased by 73 % compared with that at the start of dosing. At 60 mg/kg, etirinotecan pegol also sustained tumor growth suppression, albeit to a lesser extent compared with the higher dose level. In the ovarian tumor model, we observed no tumor growth for 4 weeks following administration of either dose of etirinotecan pegol. All animals showed decreased tumor mass, but the number of CRs increased with increasing dose. In contrast, conventional irinotecan showed only modest tumor growth delay and no regressions, and no animals survived to the planned end of the study. In addition to the weekly schedule, a single administration of 100 mg/kg etirinotecan pegol was assessed in the ovarian cancer model. Consistent with sustained tumor exposure, even the single dose provided sustained tumor growth suppression for 21 days with complete regression observed in all animals (Fig. 4f).

In summary, etirinotecan pegol was active in all tumors, with dose-related increases in the number of regressions and tumor growth delays. Median tumor growth delay was frequently the highest possible value for a study, with evidence of sustained tumor growth suppression for 2–10 weeks after administration of the last dose. Etirinotecan pegol was especially active in MCF-7 breast and NCI-N87 gastric tumors, where even the lowest dose levels showed maximal suppression of tumor growth. In contrast, conventional irinotecan at the MTD for each model resulted in little to no suppression of tumor growth.

### Etirinotecan pegol pharmacokinetics is consistent across nonclinical animal species

**Fig. 5:**
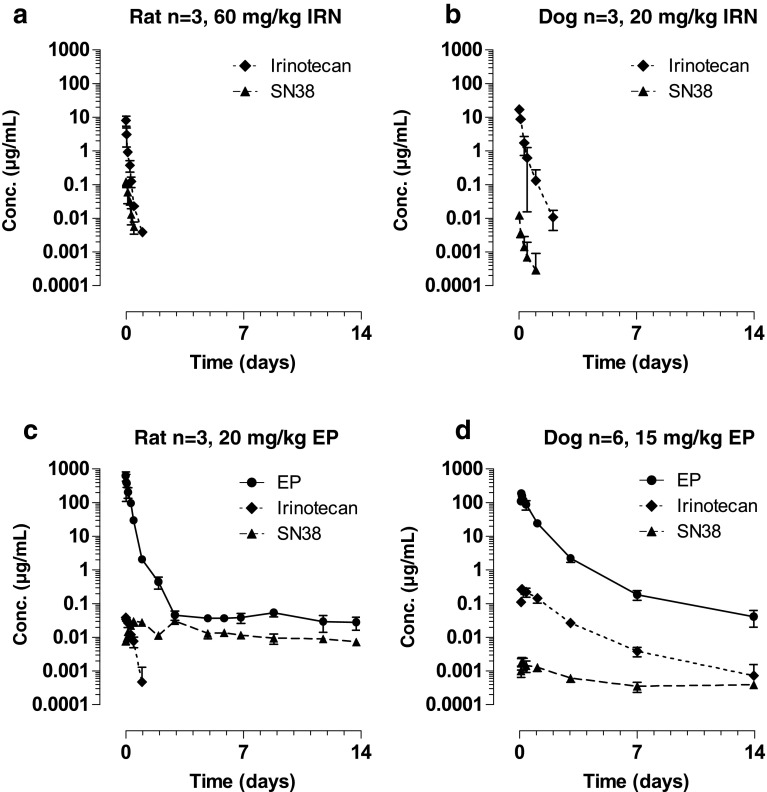
Rat and dog pharmacokinetics of etirinotecan pegol, irinotecan, and SN38 after IV administration of etirinotecan pegol or irinotecan. Irinotecan and EP were administered as 30-min (rat) or 1-hr (dog) intravenous infusions at the indicated doses

As results obtained with one animal species do not necessarily translate across different species [33], we assessed the PK of conventional irinotecan and etirinotecan pegol (including metabolites) in rats and dogs (Fig. 5). Plasma concentrations of etirinotecan pegol are sustained in all species. Plasma concentrations of the released metabolites irinotecan and SN38 are also sustained, but exposures vary across species, reflecting inherent differences in esterase activity. In all species, plasma irinotecan and SN38 *C*_max_ values after etirinotecan pegol administration are less than those after conventional irinotecan administration. Consistent with the observations in mice, plasma etirinotecan pegol concentrations remained detectable throughout the study period (14 days), while irinotecan concentrations rapidly declined to undetectable levels after administration of conventional irinotecan. As a result, SN38 was measurable during the 14-day study period after etirinotecan pegol administration, while SN38 after administration of conventional irinotecan was undetectable 24 h post-dose. The plasma half-life of SN38 was estimated to be 14 and 18 days in rats and dogs, respectively, similar to the 20-day etirinotecan pegol half-life estimated in the mouse studies. Compared with conventional irinotecan, the half-life of SN38 after etirinotecan pegol administration is 100 and 42 times longer in rats and dogs, respectively.

## Discussion

Etirinotecan pegol, a long-acting Top1 inhibitor designed to provide sustained exposure to SN38, was developed with the aim of providing increased anti-tumor activity and a better safety profile compared with short-acting Top1 inhibitors. In the nonclinical studies reported here, etirinotecan pegol outperformed irinotecan when studied at both equivalent and lower doses in a broad range of tumor models, all with different tumor growth characteristics and sensitivities to irinotecan. Animals treated with etirinotecan pegol displayed durable tumor growth suppression and marked regression, while animals treated with conventional irinotecan at MTD only exhibited temporary tumor growth inhibition as their best response. Consistent with our goal to create a long-acting Top1 inhibitor, tumor growth suppression and complete regression continued for weeks after administration of the last etirinotecan pegol dose in all tumor models, even when only a single dose was administered.

Etirinotecan pegol displayed properties previously not observed with polymeric nanoparticles, such as a circulation half-life >14 days in nonclinical species and corresponding sustained exposure to irinotecan and SN38. These characteristics resulted in a gradual decline in plasma etirinotecan pegol concentration over time, yielding a 300-fold increase in the exposure (measured by AUC) of etirinotecan pegol in mouse plasma that could distribute to the tumor compared with conventional irinotecan. Furthermore, elimination of etirinotecan pegol from the tumor was even slower compared with plasma, resulting in concentrations 2 to 50 times higher in the tumor compared with plasma for >90 % of the study period. This extent and duration of localization in tumor is consistent with the enhanced permeation and retention (EPR) effects demonstrated for macromolecules [34, 35]. Tumor localization of etirinotecan pegol via EPR is pronounced and sustained, likely benefiting from the prolonged circulation in plasma, which promotes time-dependent extravasation through the leaky tumor microvasculature [36, 37], as well as the passive trapping of the high molecular weight polymer.

Etirinotecan pegol and conventional irinotecan are both prodrugs that require conversion to SN38 for activity. With simultaneous PK/PD modeling of control, irinotecan, and etirinotecan pegol treatments, we confirmed that tumor growth suppression was related to the extent and duration of tumor SN38 exposure. Etirinotecan pegol that accumulated in tumor tissue most likely served as a reservoir for continued release of SN38 in the tumor tissue. The increased and sustained exposure of tumor tissue to SN38 from etirinotecan pegol resulted in greatly increased and sustained tumor growth suppression compared with conventional irinotecan. These PK/PD studies were the first to include PK monitoring of etirinotecan pegol, and we elected to use the same dose and schedule commonly used for conventional irinotecan in mouse models of human tumors. The resultant tumor SN38 concentration–time profiles after etirinotecan pegol treatment showed substantial accumulation beyond that needed to inhibit tumor growth. However, by simultaneously fitting the PK/PD model to tumor SN38 concentration and relative tumor volume versus time data from all treatment groups, it was possible to estimate tumor *EC*_*50*_ values. These values can be used to guide selection of dose and schedule in subsequent studies. In fact, administration of a single dose of etirinotecan pegol was explored in a mouse activity study using the A2780 ovarian cancer model, which was conducted after PK/PD results were obtained. Consistent with the PK/PD results, the single dose was as efficacious as three weekly administrations of etirinotecan pegol.

Simulations using the HT29 tumor PK/PD model indicated that administration of an IV infusion of 240 mg/kg/day conventional irinotecan for 60 days would be required to achieve SN38 tumor exposure comparable to that observed with the 40-mg/kg etirinotecan pegol q4d×3 regimen. This required daily dose of irinotecan is twice its single-dose LD_10_ in mice of ~120 mg/kg and would thus be expected to cause significant drug-related mortality. In contrast, 90 mg/kg of etirinotecan pegol administered q4d×3 to mice with HT29 tumors was well tolerated, with only 5 % loss in mean body weight.

Our studies also show that etirinotecan pegol and SN38 derived from etirinotecan pegol remain in the plasma of rats and dogs for at least 14 days. In mice, etirinotecan pegol enhanced SN38 exposure in solid tumors, thereby improving anti-tumor activity. The sustained exposure to etirinotecan pegol and SN38 described here in different animal species was also observed in human cancer patients. Following administration of etirinotecan pegol, the elimination *t*_1/2_ was 21 days for etirinotecan pegol and 50 days for SN38 [38]. The etirinotecan pegol half-life is approximately 36 times longer than the half-life for conventional irinotecan and represents a similar increase to that observed in nonclinical species. Several polymeric conjugates of irinotecan or SN38, all intended to improve the PK properties of irinotecan or SN38, are being studied clinically. They include: MM-398, a liposomal formulation of irinotecan; ILH-305, a PEGylated liposomal formulation of irinotecan; EZN-2208, a PEG conjugate of SN38; and NK-012, a copolymer consisting of PEG and partially SN38-bound polyglutamate. All conjugates have reported activity in nonclinical models compared with conventional irinotecan [39–42]; however, they have varied effects on SN38 exposure in patients, as reflected by SN38 half-lives of 75 h reported for MM-398 [43], 20 h for EZN-2208 [44], 209 h for NK-012 [45], and undetectable SN38 levels by 96 h post-dose for ILH-305 [46]. The sustained exposure observed with etirinotecan pegol in cancer patients was associated with promising activity during both Phase 1 [38] and Phase 2 [47, 48] studies of etirinotecan pegol. In particular, patients with third-line metastatic breast cancer of all types (including triple-negative disease) who received etirinotecan pegol demonstrated a confirmed objective response rate of 29 % by RECIST criteria [48]. The advantages imparted by the polymer employed in etirinotecan pegol that are associated with superior activity and improved PK in the animal species thus translate well to the clinical setting, leading to the first long-acting Top1 inhibitor in Phase 3 clinical development.

In conclusion, etirinotecan pegol provides sustained SN38 exposure in mouse xenograft tumors and increased anti-tumor activity compared with short-acting, conventional irinotecan. Our data show that prolonged circulation time and tumor localization mediated by the polymer moiety in etirinotecan pegol result in increased tumor exposure to SN38. Furthermore, the favorable changes in plasma and tumor SN38 exposures following etirinotecan pegol dosing correlate well with superior suppression of tumor growth compared with conventional irinotecan. The anti-tumor activity observed in these preclinical studies support continued development of etirinotecan pegol for the treatment of a wide variety of tumors.

## Electronic supplementary material

Below is the link to the electronic supplementary material.

Supplementary material 1 (PDF 1117 kb)
