# Supplementary material for: Nonclinical pharmacokinetics and activity of etirinotecan pegol (NKTR-102), a long-acting topoisomerase 1 inhibitor, in multiple cancer models
Source: Cancer Chemother Pharmacol. 2014 Sep 17;74(6):1125–37. doi: 10.1007/s00280-014-2577-7 (PMC4236622; doi:10.1007/s00280-014-2577-7)
Supplement: Supplementary file 1 — Supplementary material 1 (PDF 1117 kb) [file 280_2014_2577_MOESM1_ESM.pdf]

ELECTRONIC SUPPLEMENTARY MATERIAL

Nonclinical pharmacokinetics and activity of etirinotecan pegol (NKTR-102), a long-acting topoisomerase 1 inhibitor, in multiple cancer models

Ute Hoch[1], Carl-Michael Staschen[1], Randall K. Johnson[2], Michael A. Eldon[1]

[1]Nektar Therapeutics, San Francisco, CA, USA

[2]Food and Drug Administration, Rockville, MD, USA

Corresponding author:

Ute Hoch, Ph.D.

Nektar Therapeutics: 455 Mission Bay Boulevard South, San Francisco, CA 94158, USA

Tel: (415) 482-5635; fax (415) 339-5382; email: uhoch@nektar.com

Electronic Supplementary Material – Figure Legends

Online Resource 1 Schematic of tumor PK/PD model

Online Resource 2 Observed and model-predicted plasma and tumor concentration-time profiles of analytes after intravenous administration of three doses (Days 0, 4, 8) of irinotecan and etirinotecan pegol to NCI-H460 tumor-bearing mice

a, b, After administration of conventional irinotecan, plasma (a) and tumor (b) irinotecan concentrations rapidly declined to below measurable concentrations within 12 hrs of dosing. **c,** After administration of etirinotecan pegol, plasma etirinotecan pegol concentrations also declined rapidly; however, the decline was less rapid than that observed for irinotecan, and concentrations remained measurable throughout each dosing interval and for the duration of the study. **d,** In contrast to plasma, etirinotecan pegol tumor concentrations continued to accumulate with each dose, reached a maximum after the last administration, and was followed by a slow decline. Starting 24 hrs after each dose, etirinotecan pegol concentrations in the tumor exceeded those in the plasma, consistent with tumor targeting through the enhanced permeation and retention effect. N=4 animals/timepoint. Etirinotecan pegol and irinotecan were administered as an intravenous bolus at 40-mg/kg irinotecan equivalents. Symbols, mean concentration values; solid lines, model-predicted concentration values.

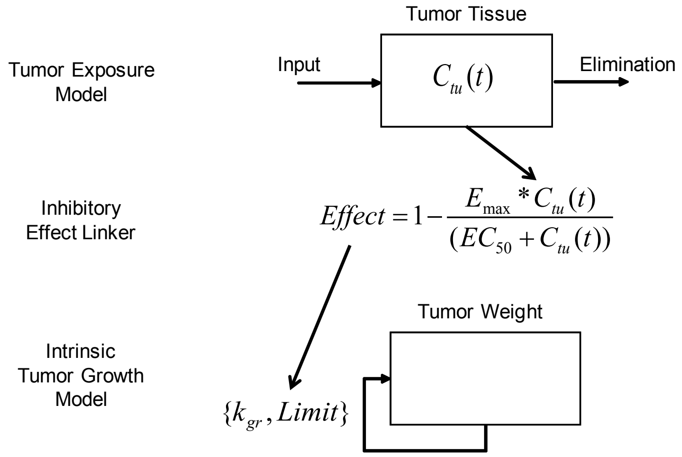

Hoch et al, Online Resource 1

Conventional Irinotecan Administration

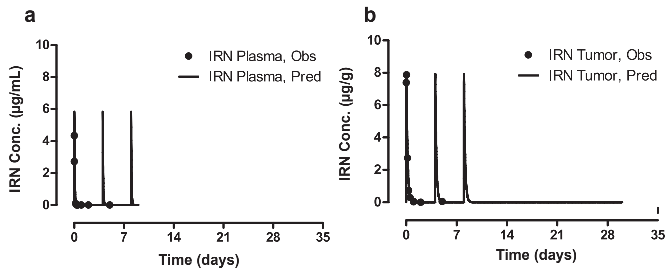

Etirinotecan Pegol Administration

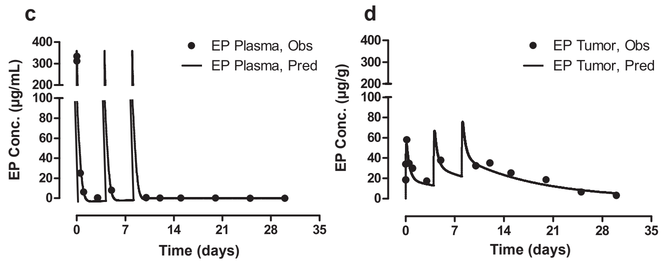

Hoch et al, Online Resource 2
